# OCT1 (SLC22A1) transporter kinetics and regulation in primary human hepatocyte 3D spheroids

**DOI:** 10.1038/s41598-024-67192-6

**Published:** 2024-07-27

**Authors:** Evgeniya Mickols, Alina Hartwig, Niklas Handin, Malin Stüwe, Jens Eriksson, Jakob Rudfeldt, Kristin Blom, Mårten Fryknäs, Mikael E. Sellin, Volker M. Lauschke, Maria Karlgren, Per Artursson

**Affiliations:** 1 Department of Pharmacy, Uppsala University, Uppsala, Sweden; 2 Science for Life Laboratory, Department of Medical Biochemistry and Microbiology, Uppsala University, Uppsala, Sweden; 3 Department of Medical Sciences, Division of Cancer Pharmacology and Computational Medicine, Uppsala University, Uppsala, Sweden; 4 Department of Physiology and Pharmacology, Karolinska Institute, Stockholm, Sweden; 5 Dr Margarete Fischer-Bosch Institute of Clinical Pharmacology, Stuttgart, Germany; 6 University of Tübingen, Tübingen, Germany; 7 Centre of Molecular Medicine, Karolinska Institute, Stockholm, Sweden

**Keywords:** Hepatocyte, Liver, Uptake, ASP+, OCT1, Drug transport, Drug-Drug interaction, Spheroid, 3D culture, Proteomics, Drug safety, Drug screening, Proteomics, Drug development, Fluorescence imaging, Proteomic analysis, Mass spectrometry

## Abstract

3D spheroids of primary human hepatocytes (3D PHH) retain a differentiated phenotype with largely conserved metabolic function and proteomic fingerprint over weeks in culture. As a result, 3D PHH are gaining importance as a model for mechanistic liver homeostasis studies and in vitro to in vivo extrapolation (IVIVE) in drug discovery. However, the kinetics and regulation of drug transporters have not yet been assessed in 3D PHH. Here, we used organic cation transporter 1 (OCT1/*SLC22A1*) as a model to study both transport kinetics and the long-term regulation of transporter activity via relevant signalling pathways. The kinetics of the OCT1 transporter was studied using the fluorescent model substrate 4-(4-(dimethylamino)styryl)-N-methylpyridinium (ASP+) and known OCT1 inhibitors in individual 3D PHH. For long-term studies, 3D PHH were treated with xenobiotics for seven days, after which protein expression and OCT1 function were assessed. Global proteomic analysis was used to track hepatic phenotypes as well as prototypical changes in other regulated proteins, such as P-glycoprotein and Cytochrome P450 3A4. ASP+ kinetics indicated a fully functional OCT1 transporter with a K_m_ value of 14 ± 4.0µM as the mean from three donors. Co-incubation with known OCT1 inhibitors decreased the uptake of ASP+ in the 3D PHH spheroids by 35–52%. The long-term exposure studies showed that OCT1 is relatively stable upon activation of nuclear receptor signalling or exposure to compounds that could induce inflammation, steatosis or liver injury. Our results demonstrate that 3D PHH spheroids express physiologically relevant levels of fully active OCT1 and that its transporter kinetics can be accurately studied in the 3D PHH configuration. We also confirm that OCT1 remains stable and functional during the activation of key metabolic pathways that alter the expression and function of other drug transporters and drug-metabolizing enzymes. These results will expand the range of studies that can be performed using 3D PHH.

## Introduction

Primary human hepatocytes (PHH) are integral to drug discovery, serving as a crucial in vitro model for assessing drug metabolism, toxicity, and pharmacokinetics^1–6^. When PHH are cultured in suspension or as 2D monolayers, they are short-lived and rapidly lose their drug-transporting and drug-metabolizing functions, limiting their applicability in pharmacology and toxicology^7^. Considerable efforts have been made to extend the lifespan of functional human hepatocytes. These efforts include culturing in more physiological formats such as sandwich cultures, 3D spheroid cultures, various microfluidic liver mimics or biochips, and pharmacological modification of cell signaling pathways^7–12^. We recently showed that PHH spheroids (hereafter 3D PHH) can be cultured under physiologically relevant conditions using media containing fasting glucose and insulin levels^8^. These 3D PHH maintain drug-metabolizing enzymes expression and function for weeks in the in vitro environment^8^. Similarly, they maintain the expression of clinically significant drug-transporting proteins^5,7,8^.

Organic anion transporting polypeptides (OATPs) and organic cation transporters (OCTs) transport essential drugs and nutrients into hepatocytes^13^. However, studies on the kinetics and functionality of transporters in 3D PHH are scarce and therefore warranted^14^. Here, we provide an approach for such studies using organic cation transporter 1 (OCT1), encoded by the *SLC22A1* gene, as a prototypic example.

OCT1 transports a variety of organic cations, including endogenous bioactive amines such as choline, dopamine, and thiamine; cationic xenobiotics such as the antidiabetic drug metformin, cytostatic drugs such as oxaliplatin and imatinib, the antiviral agent lamivudine, and the opioid morphine^15–20,20–22^. Therefore, the 2022 International Council for Harmonization of Technical Requirements for Pharmaceuticals for Human Use (ICH) draft guideline M12 on drug-drug interaction (DDI) studies suggests that drug candidates should be studied as substrates and/or inhibitors of OCT1 along with other hepatic transporters^23^. Notably, the expression and function of several hepatic drug transporters are altered in inflammatory conditions^24^, bile cholestasis^25^, and liver injury^26^. The expression and function of these transporters is controlled by nuclear receptors such as Farnesoid X receptor, Pregnane X receptor, Constitutive Androstane Receptor (i.e. FXR, PXR, CAR) and posttranscriptional mechanisms such as retrieval of the transporters from the cell membrane and phosphorylation by various kinases, respectively^25,27^. Therefore, we also investigated how long-term activation of such signaling pathways, some of which regulate the expression and function of other drug-transporting proteins and drug-metabolizing enzymes, affects OCT1 expression and kinetics.

## Materials and methods

### Isolation of primary human hepatocytes

Human hepatocytes were isolated from histologically normal surplus liver tissue obtained from cancer patients undergoing liver resections at the Department of Surgery at Uppsala University Hospital. The study was conducted according to the guidelines of the Declaration of Helsinki. Ethical approval has been obtained from Uppsala Regional Ethical Review Board (Ethical Approval no. 2009/028, amended 2018/1108). Donors signed informed consent in agreement with the approval from the Uppsala Regional Ethical Review Board (Ethical Approval no. 2009/028, amended 2018/1108). A previously described two-step collagenase perfusion procedure for hepatocyte isolation was performed^28^. In brief, the liver tissue was rinsed from excessive blood with Hypothermosol FRS (BioLife Solutions,WA, USA), cannulated, and perfused with collagenase and protease buffers. Cells were cryopreserved in CryoStor CS10 solution (BioLife Solutions,WA, USA) supplemented with 10% fetal bovine serum, followed by isopropanol-assisted freezing at − 80 °C for three hours and storage at − 150 °C until use.

### 3D Primary human hepatocyte spheroid culture

PHH from three donors were used in this study, and the donors' demographics and available medical history are shown in Supplementary Table 1. Hepatocyte spheroid culture was performed as previously described^8^. In brief, cryopreserved hepatocytes were gently thawed and transferred to isotonic Percoll (GE Healthcare, Chicago, Illinois) in Dulbecco's Modified Eagle Medium (DMEM) with HEPES and 4.5 g/L Glucose supplemented with fetal bovine serum (FBS; 5%), penicillin (100U/mL), streptomycin (100 µg/mL), insulin (4 µg/mL), dexamethasone (0.1 µM), and centrifuged at 100 × g for 10 min. After the centrifugation, the supernatant with cell debris and dead cells was discarded. The hepatocytes were resuspended in a warm, fully supplemented DMEM medium. PHH suspension was seeded at 5000 cells/well in Corning 4516 ultra-low attachment 384-well plates (Corning, Kaiserslautern, Germany), sedimented by 100 × g centrifugation for 10 min, and incubated at 37 °C with 5% CO_2_. The medium was gradually changed to a physiologically relevant Williams E medium without glucose, L-glutamine, phenol red and FBS (PAN-Biotech GmbH, Aidenbach, Germany) supplemented with insulin (0.58 ng/mL), glucose (990 mg/L), transferrin (5.5 µg/mL), selenium (5 ng/mL), dexamethasone (0.1 µM), penicillin (100U/mL), streptomycin (100 µg/mL) and L-glutamine (2 mM)^8^ using a Biomek 4000 Automated liquid handler. The first medium change was typically performed after the initial spheroid aggregation (within 3–5 days after the PHH seeding), starting with a 50% medium change. After that, 80% of the medium was replaced with fresh physiologically relevant serum-free William's medium at every medium change. All media and cell-culture supplements were purchased from VWR (Radnor, Pennsylvania), Thermo Fisher Scientific (Waltham, Massachusetts), or Sigma-Aldrich (Saint Louis, Missouri) unless otherwise stated.

### ASP+ kinetic assay in 3D PHH

3D PHH were used after spheroid formation (one week of culture). Prior to the fluorophore 4-(4-(dimethylamino)styryl)-N-methylpyridinium (ASP+) uptake assay, PHH culture medium was exchanged to warm Hank's balanced salt solution (HBSS). The PHH plate was centrifuged at 100 × g for 30 s and incubated at 37 °C in the HBSS for 10 min. Next, HBSS containing ASP+ was added, giving a final ASP + concentration of 1–60 μM. The plate was centrifuged at 100 × g for 30 s, and fluorescence from ASP + in each spheroid was measured at regular intervals for up to 60 min and performed in at least three spheroids each. The wavelengths were set to λ_ex_ = 475 nm and λ_em_ = 605 nm in a Tecan Spark (Männhedorf, Switzerland) or a CLARIOstar microplate reader (BMG Labtech, Ortego, Germany). The gain adjustment setting was kept off to ensure the comparability of the data when several culture plates were used in the assay. Importantly, only centrally placed spheroids give a correct readout in the fluorescent assay. Hence, peripherally located spheroids were excluded throughout this study.

OCT1 kinetics calculations were performed as previously described for SLC transporters^29^. To assess the uptake kinetics of ASP+ , the initial uptake rate (0–19 min) was obtained from the linear regression equation and was plotted against the ASP+ concentration (1–60 µM). K_m_ and V_max_ values were determined by non-linear regression according to the Michaelis–Menten Eq. (1) using Prism version 9 (GraphPad, San Diego, CA), where V is the uptake rate, V_max_ is the maximal uptake rate (at saturating substrate concentration), [S] is the substrate concentration, K_m_ is the substrate concentration at which the uptake rate is half of V_max_.

1 $$v=\frac{{V}_{max} S}{{K}_{m}+ S}$$

### ASP+ Uptake studies in individual 3D PHH spheroids

Live fluorescence microscopy was performed on a modified Nikon Eclipse Ti-2 microscope. The microscope was equipped with a photometric prime 95B camera, and images were taken through a Nikon 10X/0.45 Plan Apo Lambda objective using a 1.5X Optovar. The PHH plate was kept at 37 °C and 5% CO_2_ during the data acquisition. A 475/28 Lumencore Spectra-X bandpass filter was used for excitation, while the emission was collected through a Chroma HQ610/75 nm filter. Image processing was performed in Fiji/ImageJ^30^.

### OCT1 Inhibition assay

The inhibition assay was performed based on a previously published method^31^. First, the intrinsic fluorescence of the OCT1 inhibitors was measured to ensure a lack of interference with the ASP+ assay. Before the inhibition experiments, the culture medium was changed to HBSS containing none or one of the inhibitors, namely ketoconazole, verapamil, clomipramine, diltiazem, clotrimazole or chlorpromazine to give a final concentration of 100 µM. The plate was centrifuged at 100 × g for 30 s and incubated at 37 °C for 10 min. Next, HBSS with the corresponding inhibitor (100 µM) and ASP+ was added to a final ASP+ concentration of 1 µM and the plate was centrifuged at 100 × g for 30 s. The fluorescence was measured as described in the section above. Fluorescence read out from the 3D PHH incubated only with ASP + was used to calculate the inhibitory potential of the compound tested. The inhibition was calculated as a percentage of the maximal ASP + uptake rate. All experiments were performed in triplicates in two or three independent experiments.

### Long-term exposure of 3D PHH to xenobiotics

First, a literature search was performed to identify compounds which could modulate the expression and function of the OCT1 transporter (Table 2). Following complete spheroid formation, the PHH culture medium was exchanged for culture medium supplemented with one of the selected compounds. The compound exposure concentrations were determined using the approach developed by Vorrink et al.^32^. In brief, therapeutic exposure concentrations (C_max_) or equal were obtained from the literature and PHH spheroids were exposed to the one and five times clinical C_max_ concentrations of the individual compounds. The 3D PHH were incubated with the compounds for seven days, and the culture medium was changed every 48–72 h to ensure the replenishment of the nutrients and the potential OCT1 modulators. Most compound stock solutions were prepared in dimethyl sulfoxide (DMSO) and subsequently diluted in the serum-free cell-culture medium to a final DMSO concentration of 0.025%. The paracetamol stock was dissolved in HBSS and tumor necrosis factor-alpha (TNF-α) was reconstituted in phosphate-buffered saline (PBS). After one week incubation time with selected compounds, ASP+ uptake (10 µM) was measured as described above in section "ASP+ kinetic assay in 3D PHH". Briefly, ASP+ concentration was kept constant (10 µM) and the initial uptake rate (0–19 min) was obtained from the linear regression equation and normalized to the initial uptake rate of control 3D PHH.

### PHH viability assay

PHH viability and energy state were assessed using the CellTiter-Glo® 3D assay (Promega, Madison, Wisconsin) according to the manufacturer's instructions. Briefly, the PHH plates and CellTiter-Glo® 3D reagent were equilibrated to room temperature for approximately 30 min. Subsequently, CellTiter-Glo® 3D reagent was added to the well in a volume equal to the cell culture medium, and the plate was incubated at the orbital shaker for approximately 30 min more. The luminescence was measured in a Spark plate reader (TECAN) for a minimum number of four technical replicates/wells.

### Global proteomics analysis

#### Sample preparation procedure

PHH spheroids (at minimum 40 spheroids per condition) were collected in low protein binding microcentrifuge tubes (Eppendorf, Fisher Scientific, Gothenburg), washed twice with ice-cold PBS and snap-frozen in liquid nitrogen. Next, samples were rapidly defrosted and lysed in 25 mM HEPES buffer, pH 7.6, containing 4% sodium dodecyl sulfate and 1 mM DTT, and proteins were denatured at 95 °C for 5 min. The sample preparation procedure was performed using a modified single-pot, solid-phase-enhanced sample-preparation (SP3) technology^33^. In brief, samples were sonicated, and proteins were alkylated, bound to paramagnetic beads and digested overnight with trypsin and LysC digestion enzymes. The following day the peptide digests were cleaned up on the beads and subsequently released by change in hydrophobicity, transferred to glass LC–MS vials and dried in a Speedvac at 40 °C. Protein and peptide concentrations were determined by fluorometric quantification with the Protein Broad Range Assay on the Qubit 4 Fluorometer (Thermo Fisher Scientific, Waltham, MA).

#### LC–MS/MS analysis of the peptides

Dried peptides were reconstituted in 0.1% formic acid in LC–MS/MS grade water. Peptides were separated on an EASY-spray C_18_-column (50 cm, 75 μm inner diameter), using an acetonitrile/water gradient (0.1% formic acid) at 300 nL/min. Eluted peptides were analyzed using the TopN method (full MS followed by ddMS2 scans) on an Orbitrap Q Exactive HF mass spectrometer (Thermo Fisher Scientific), operating in a data-dependent mode with survey scans at a resolution of 120,000, AGC target of 3 × 10^6^ and maximum injection time of 120 ms. The top 15 most abundant isotope patterns were selected from the survey scan with an isolation window of 1.7 m/z and fragmented with nCE at 26. The MS/MS analysis was performed with a resolution of 30,000, AGC target of 1 × 10^5^ and a maximum injection time of 50 ms. Each sample was injected in triplicate. The mass spectrometry proteomics data have been deposited to the ProteomeXchange Consortium via the PRIDE partner repository with the dataset identifier PXD045733^34,35^.

#### Data analysis

The raw MS datafiles were processed using MaxQuant version 2.4.2.0^36^. Proteins were identified by searching MS and MS/MS data of peptides against a reference human proteome database retrieved from the UniProtKB/Swiss-Prot curated database on 2023-07-13^37^. A detailed description of the parameters used for peptide identification and integration by MaxQuant can be found in the mqpar.xml uploaded to PRIDE. Briefly, carbamidomethylation was set as fixed modification and oxidation and acetylation as variable modifications; match between runs (MBR) and label-free quantification (MaxLFQ) algorithms were used. The decoy sequences were created by reversing the target sequences. Peptide-spectrum match (PSM), peptides and proteins were validated at a 1% false discovery rate (FDR) estimated using the decoy hit distribution. Quality control of the MaxQuant search was performed using an R-based quality control pipeline called Proteomics Quality Control (PTXQC) version v1.0.16^38^.

Subsequent data clean-up was performed using an in-house developed proteomics data pipeline in R version 4.3.0. Concisely, ProteinGroups data table was cleaned up using Tidyverse package, and label-free quantification (LFQ) intensities were normalized using variance stabilization normalization (Vsn) using vsn package (Bioconductor 3.17 release)^39,40^. Then protein abundances (fmol/μg total protein) were calculated with the Total Protein Approach (TPA) using the mean intensity from the three technical replicates^41^. The ADME-related gene list was used for subsetting of proteins of interest^42^. If biological replicates were available, median protein abundances were calculated. Data overview analysis was performed in Perseus version 2.0.10.0^43^. Gene ontology (GO) enrichment analysis was performed using GO Enrichment analysis and visualization tool Gorilla^44^. Differential expression analysis DEqMS was implemented via web-platform Amica^45,46^.

### Quantification and statistical analysis

Unless otherwise stated, statistical analysis and plot generation were carried out using GraphPad Prism version 9.0.0 (GraphPad Software, San Diego, CA). All results are presented as mean values ± SD of one to three biological replicates with at least three technical replicates if not otherwise specified.

### Ethical approval

The study was conducted according to the guidelines of the Declaration of Helsinki. Tissue donors signed an informed consent, under the ethical approval from Uppsala Regional Ethical Review Board (Ethical Approval no. 2009/028, amended 2018/1108). Informed consent and ethical approval (both in Swedish) could be provided to reviewers upon request.

## Results

### OCT1 expression in hepatocytes and probe substrate selection

**Figure 1:**
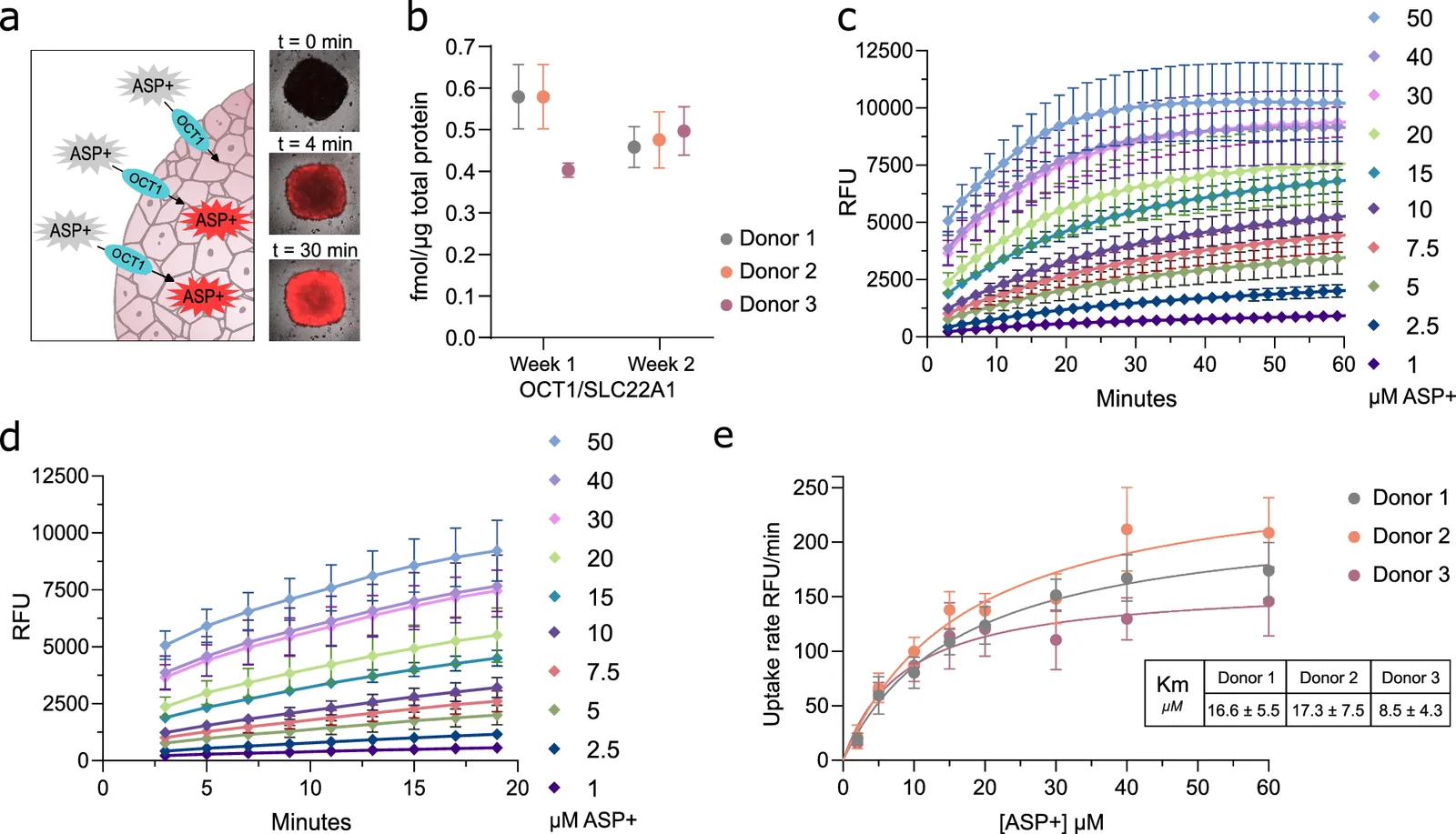
OCT1 expression in 3D PHH and uptake kinetics of ASP+ . (**a**) Schematic representation of the ASP+ uptake mechanism and fluorescence microscopy visualization. (**b**) Expression of OCT1 transporters in primary human 3D hepatocyte spheroids from 3 donors after one and two weeks of culture. Protein expression is in fmol/µg total protein and was measured in 3 technical replicates. The expression levels between weeks and across PHH donors were not significantly different when evaluated with differential expression analysis for global proteomics using DEqMS. (**c**) Time-dependent fluorescence was measured for different ASP+ concentrations (0 to 50 µM) and followed for 60 min in 3D PHH from donor 1 (n = 4–6). Standard deviations counted as a percent of ASP+ uptake remained stable throughout all concentrations (13 to 15%). (**d**) Linear uptake interval (0 to 19 min) was used to calculate K_m_ and V_max_ in 3D PHH from donor 1 (n = 4–6). (**e**) Michaelis–Menten kinetics for each of the three 3D PHH donors in Table 1. The points represent the mean uptake rate, and the error bars show the standard deviation of uptake rate per point, n = 8–12.

The cationic fluorophore ASP+ has already been used in studies of OCT1 uptake and inhibition in various cell types and conventional 2D hepatocyte cultures^31,47,48^. ASP+ shows binding-associated fluorescence in the membranes of living cells but not in cell culture media or common buffer systems^49,50^. This facilitated the development of an assay for quantifying the kinetics of the transporter, in which the fluorescence signal is proportional to the amount of ASP+ taken up by hepatocytes in 3D spheroids (Fig. 1a).

ASP+ is a common substrate of several SLC transporters such as OCT1, 2, and 3 (SLC22A1, SLC22A2, SLC22A3), as well as OCTN1 and 2 (SLC22A4, SLC22A5), and MATE1 (SLC47A1)^51–53^. Even if not all of these transporters are normally expressed in the human liver, we examined the expression of these proteins in the 3D PHH. After one week of culture, we observed no significant difference in OCT1 expression between liver donors (Fig. 1b) with comparable levels as to what was previously published^8^. OCT1 expression in spheroids cultured for two weeks was largely similar. At the same time, OCT2, OCT3, OCTN1, OCTN2 and MATE1 were not detected by global proteomic analysis (Data S1. Protein concentrations), consistent with expression patterns reported in the literature for these transporters^8,54,55^. Thus, we conclude that OCT1 is the dominant ASP+ uptake transporter expressed in our 3D PHH model.

### Transport kinetics of ASP+ in primary human hepatocyte spheroids

Time- and concentration-dependent uptake of ASP+ was studied to determine transport kinetics. ASP+ was rapidly taken up in 3D PHH during the first 20 min, and the uptake rate subsequently decreased until it gradually reached saturation (Fig. 1c). Using the initial linear uptake rate (Fig. 1d and Supplementary Fig. 1), we obtained Michaelis–Menten constants (K_m_) for all three tested hepatocyte donors (Fig. 1e; Table 1). The V_max_ values were determined by extrapolating the curve to the point of the maximal reaction rate. The obtained apparent K_m_ and V_max_ values ranged between 8.53 and 17.20 µM and 161.6–271.5 RFU/min, respectively (Table 1), similar to those previously reported in the literature^47,50^. Thus, no significant interindividual variability was observed between the three donors. This suggests that interindividual variability does not have a major impact on the transport kinetics determined in this specific study. However, donor dependent variability should generally be taken into consideration when working with PHH in different configurations^48,56^.

**Table 1:** Apparent K_m_ and V_max_ values for three 3D PHH donors.

Donor	Apparent K_m_, µM		Apparent V_max_, RFU/min	
	Best-fit value	95% CI	Best-fit value	95% CI
1	16.60	From 11.93 to 23.05	228.8	202.9–261.7
2	17.29	From 11.35 to 26.50	271.5	232.5–325.4
3	8.53	From 5.184 to 13.85	161.6	140.3–189.4

### OCT1 inhibitors reduce ASP+ uptake in 3D PHH spheroids

**Figure 2:**
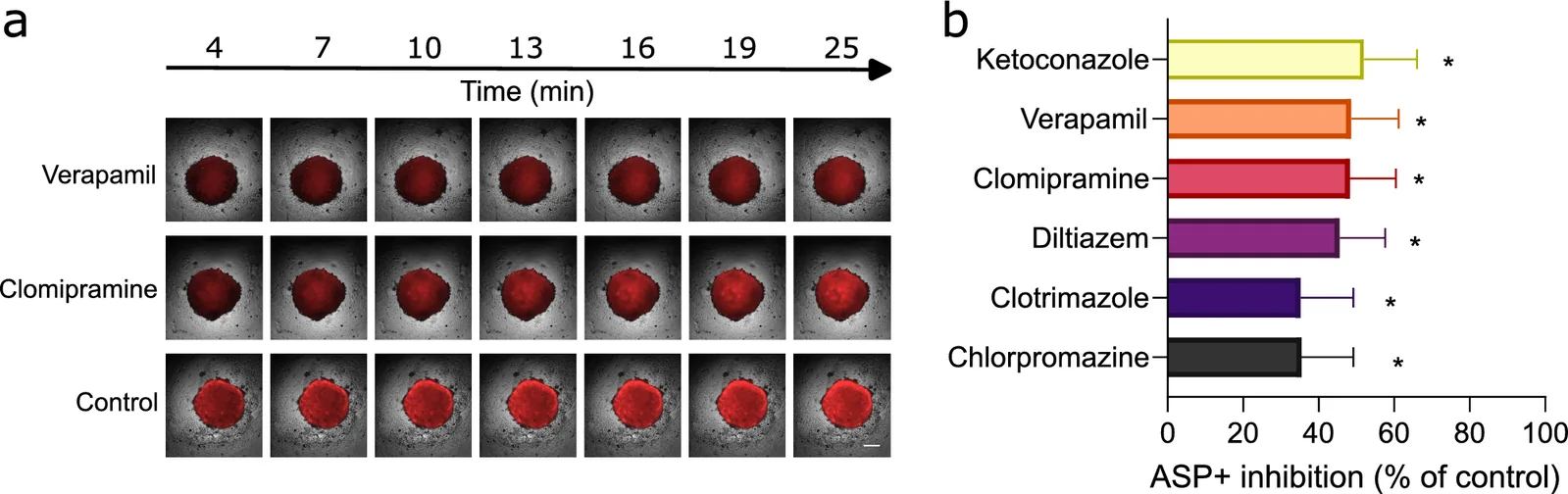
OCT1 inhibitors reduce ASP+ uptake in 3D PHH spheroids. (**a**) ASP+ (1 µM) uptake alone and in the presence of verapamil (100 µM) and clomipramine (100 µM) in 3D PHH cultured for one week. Numbers indicate ASP+ incubation time in minutes. Scale bar = 200 μm. (**b**) Uptake of ASP+ (1 µM) was measured after 5 min in the presence of 100 µM of the OCT1 inhibitors. The inhibitory effect of compounds is shown as the percentage of vehicle control, n = 8–12. All observations were statistically significant in multiple unpaired t-tests compared to vehicle control.

Inhibition of ASP+ uptake was examined with six well-established OCT1 inhibitors of various chemical and physical properties under Lipinski's rule of five (Supplementary Table 2)^31,57^. ASP+ inhibition was observed in individual spheroids using live microscopy (Fig. 2a) as well as the 384-well plate reader (Fig. 2b and Supplementary Table 3). ASP+ uptake in the presence of inhibitors was reduced to 35–52% of uptake observed in controls already after 5 min. Only minor additional inhibition of 2–7% was observed after 25 min (Supplementary Fig. 2 and Supplementary Table 3).

### Long-term exposure to OCT1 modulators

Next, we investigated the sensitivity of OCT1 to long-term drug exposure. To this end, we exposed spheroids to compounds that potentially affect OCT1 transporter expression and function through various mechanisms of action (Table 2). The effects of all compounds were examined at one and five times Cmax or equivalent for tumor necrosis factor alpha (TNF-α) or free fatty acids (FFA)^24,58,59^. After seven days of exposure to the compounds using donor 1 3D PHH, we examined ASP+ uptake to assess transporter kinetics and ATP content as a marker of spheroid viability.

**Table 2:** Compounds investigated for effects on OCT1 transporter expression and function.

Group		Compound	In vivo Cmax or equal
Nuclear receptor modulators	PXR, FXR	T0901317^60^	10 µM^61^
	PXR	Rifampicin^15,62^	9.96 µM^32^
	FXR	Chenodiol^63,64^	10.0 µM^64^
	PPARα/δ	Elafibranor^65^	1.71 µM
Drug-Induced Liver Injury		Chlorpromazine^7^	0.84 µM^32^
		Paracetamol^32^	136 µM^32^
		Diclofenac^32^	9.43 µM^32^
Chronic inflammation		TNF- α^24^	1 ng/mL^24^
Steatosis		FFA^66^	320 μM^58^

**Figure 3:**
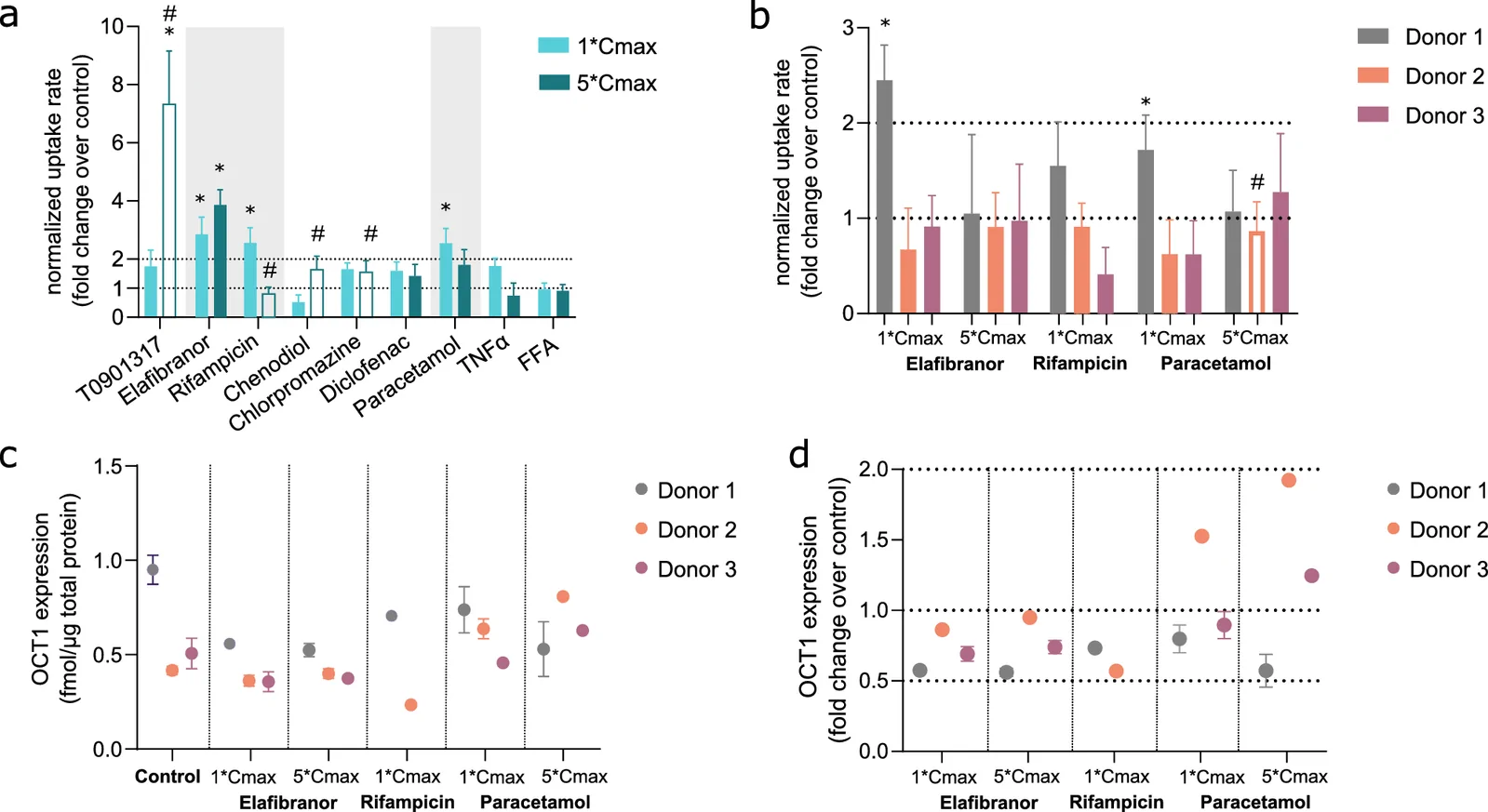
Long-term exposure of 3D PHH to OCT1 modulators. (**a**) Normalized uptake of ASP+ (10 µM) after one week exposure to one and five times Cmax of the modulators using 3D PHH from a single donor (donor 1). Bars show mean ASP+ uptake normalized to untreated control, and error bars indicate standard deviation, n = 4–9. The open staples and # indicate cell viability of less than 80% (see also Supplementary Fig. 3). Compounds highlighted in grey were selected for further evaluation in multiple PHH donors. (**b**) Normalized uptake of ASP+ (10 µM) after one week of exposure to 1 and 5 times Cmax of xenobiotics in three different PHH donor batches (5 × Cmax rifampicin exposure was excluded due to toxicity of the compound). Statistical significance was assessed in two-way ANOVA analysis with Tukey's multiple comparison test. Dotted lines indicate control level and two-fold change from control (n = 8–15). (**c**) OCT1 transporter expression in 3D PHH spheroids from three donors after exposure to modulators. Protein expression is presented in fmol/µg total protein and as an average value between biological replicates. In donor 3, 1 × Cmax condition, OCT1 was not detected. (**d**) Normalized OCT1 transporter expression after exposure to 1 and 5 times Cmax of xenobiotics, normalized to expression in vehicle control.

While most compounds had no effect on ASP+ uptake in donor 1, a moderate but significant increase in ASP+ uptake was observed in 3D PHH donor 1 for four compounds, namely: T0901317 (5 × Cmax): 7.3 ± 1.8-fold; rifampicin (1 × Cmax): 2.5 ± 0.5-fold; elafibranor (1 × and 5 × Cmax): 2.9 ± 0.3-fold and 3.8 ± 0.5-fold, respectively; and paracetamol (1 × Cmax): 2.5 ± 0.5-fold (Fig. 3a). However, the viability of 3D PHH exposed to T0901317 (5 × Cmax) was low (≤ 80%) compared with control 3D PHH cultured with vehicle alone, indicating a potential false positive result (Supplementary Fig. 3). High concentrations of TNF-α or FFA, previously demonstrated to transform 3D PHH into in vitro models of inflammation and steatosis, respectively, also did not affect ASP+ uptake by spheroids. Similarly, chenodiol, chlorpromazine and diclofenac did not significantly alter ASP+ uptake compared with vehicle controls. Thus, only a small effect of modulators on OCT1 activity in donor 1 was observed.

The compounds that produced a significant change in ASP+ uptake at nontoxic concentrations in Donor 1, i.e., elafibranor (1 × and 5 × Cmax), rifampicin (1 × Cmax), and paracetamol (1 × and 5 × Cmax), were selected for further study in all three donors. The 3D PHH were treated with the compounds for 1 week. We again observed that in donor 1, exposure to elafibranor and paracetamol moderately increased ASP+ uptake in these spheroids. However, in cultures from donors 2 and 3, we did not observe significant changes in ASP+ uptake (Fig. 3b). Interestingly, donor 2 was sensitive to high concentrations of paracetamol (27% viability, Supplementary Fig. 4), whereas 3D PHH from donors 1 and 3 showed no or minor change in viability when exposed to paracetamol. Furthermore, using global proteomics, we found that the expression of OCT1 in primary human hepatocytes remained stable (within twofold from control level, considering given biological variability between donors) at the resolution achieved by this method (Fig. 3c). When normalized to OCT1 abundance in vehicle control, the OCT1 expression change remained within twofold change (Fig. 3d)^8,62^.

### Global proteomics analysis across hepatocyte donors after long-term exposure to xenobiotics

**Figure 4:**
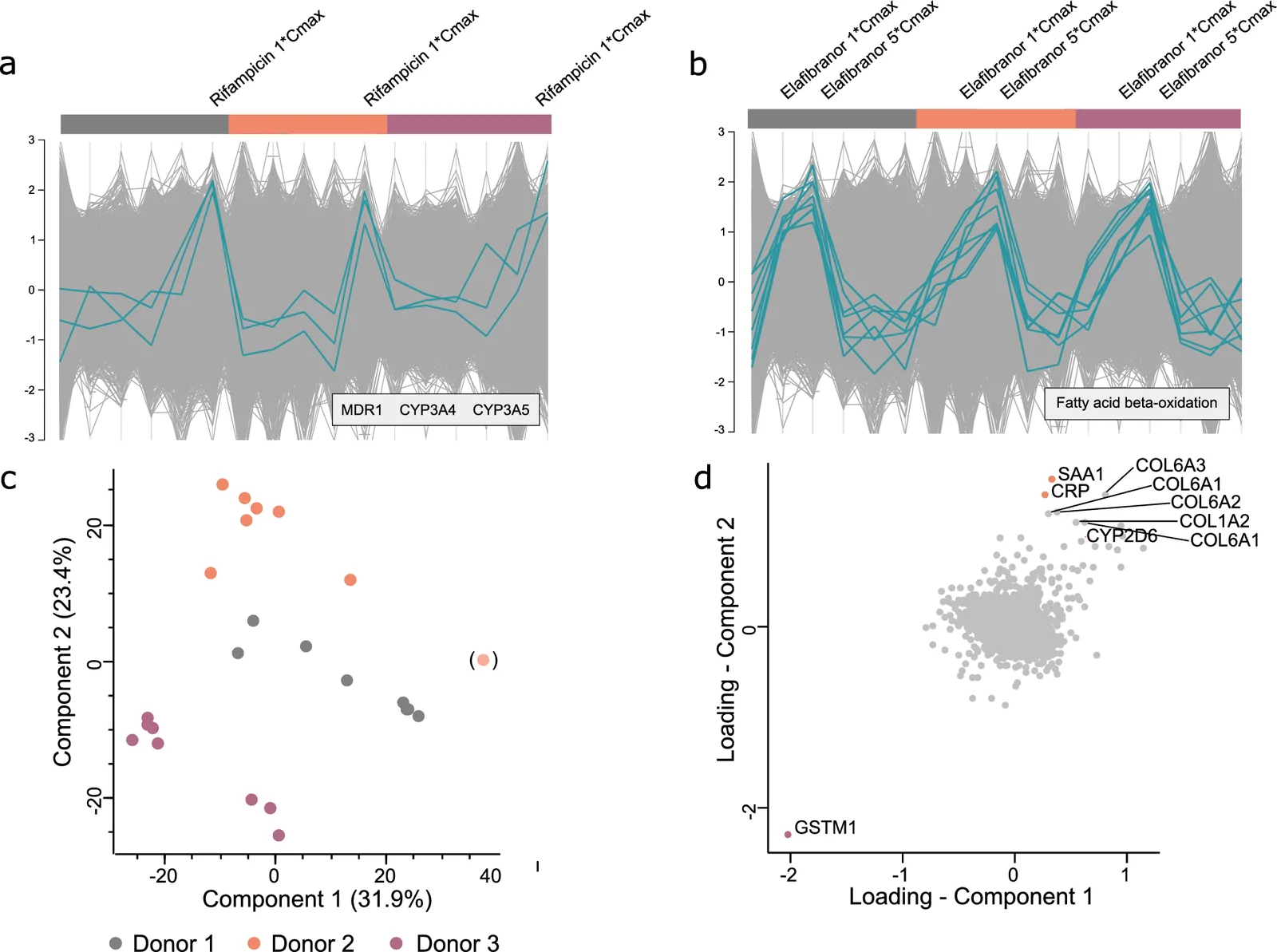
Global proteomic profiling of 3D PHH. (**a**) Profile plot of proteins upregulated in 3D PHH exposed to rifampicin and (**b**) elafibranor. The Y-axis represents the z-score normalized protein expression across the samples. (**c**) Principal component analysis of global proteomes. The number in parentheses is the percentage of variability explained by each component. The dot in parentheses represents donor 2 at 5 × Cmax paracetamol exposure and was excluded from further analysis due to poor protein identification rate. A total of 3533 proteins were identified, of which 2875 could be quantified. (**d**) Loadings of PCA (panel c), the proteins contributing most to the variance are highlighted.

An alternative explanation for the modest effects on OCT1 function and expression could be that the 3D PHH did not respond to long-term exposure. Therefore, we looked for prototypical changes in ADME proteins caused by the compounds. Importantly, we found compound-specific changes in the proteomes. The PXR agonist rifampicin is a known CYP3A4/5 and MDR1 inducer, and we confirmed that after the exposure to rifampicin, these proteins were upregulated in 3D PHH from each of the three donors (Fig. 4a)^62^. In addition, elafibranor treatment led to significant upregulation of proteins from the fatty acid beta-oxidation pathway (1.8*10^–3^ q-value; Fig. 4b), consistent with previously published data^67–69^. Paracetamol exposure induced stochastic changes in the proteomes, and no particular pathway was enriched when 3D PHH were exposed to therapeutic concentrations of this compound (Supplementary Table 5, Supplementary Fig. 6). These results suggest that 3D PHH responds to the test compounds as predicted, while OCT1 levels remain largely refractory to such stimuli.

After verifying that the 3D PHH had the expected functionality, we next used principal component analysis (PCA) to obtain a global view of the effects of donor and drug exposure. We found that proteomes differed considerably between liver donors, with the first component explaining 31.9% of the variance and the second component explaining 23.4% of the variance (Fig. 4c). Hierarchical clustering supported this conclusion in a transparent manner (Supplemetary Fig. 5). Next, we examined the proteins that contributed most to distinguish the proteomes by looking at the principal component loadings. Consistent with PCA, hierarchical clustering, and donor profile plots (Supplementary Fig. 6 and Supplementary Table 6), the differentially expressed proteins included type I collagens that were less abundant in donor 2 and collagens VI that were upregulated in the same donor; as well as glutathione S-transferase Mu 1 (GSTM1), which was found only in 3D PHH of donor 3; CYP2D6 (a well-know polymorphic enzyme), which was less abundant in donor 2; and finally, C-reactive protein (CRP), which was upregulated in donor 2 (Fig. 4d). Interestingly, donor 2 also had elevated levels of complement- and lipopolysaccharide (LPS)-binding proteins (Supplementary Fig. 6 and Supplementary Table 6), which most likely contributed to this donor being more distant from the other donors in the hierarchical cluster analysis in (Supplemetary Fig. 5).

Additionally, we looked more closely at the ADME-related proteins and again found that the proteins were clustered based on the donor variable^42^. We identified 30 clinically important proteins and tracked the expression of these proteins across PHH donor and exposure conditions (Data S1. Protein concentrations, Supplementary Fig. 7). Except for CYP3A4/5 and MDR1 induction by rifampicin described above, we were unable to correlate exposure conditions with the induction or inhibition of the other identified ADME proteins.

In summary, in agreement with previously published data, global proteomics analysis of 3D PHH highlights that donor-to-donor biological variability, particularly in ADME enzymes, contributes to the dataset variability^70^. However, OCT1 protein levels appear essentially stable across donors and various stimulations.

## Discussion

Solute transporting proteins (SLCs) play a critical role in cellular homeostasis by facilitating the translocation of ions and molecules, such as nutrients and metabolites, across cell membranes, thus maintaining cellular homeostasis^14^. SLC-mediated transport is particularly important in the liver, where SLCs in the plasma membrane of hepatocytes, such as OCT1, transport important vitamins and drugs including thiamine and metformin^17,19,26^. While the importance of SLCs such as OCT1 has been studied in suspension and 2D cultures^31,47,48^, their kinetics in multilayered 3D cultures are less trivial to characterize due to the three-dimensional culture set up. In this study, we investigated the kinetics of the OCT1 substrate ASP+ in 3D PHH. Since these spheroids maintain a non-proliferating, differentiated phenotype for weeks in culture in agreement with the long half-life of mature hepatocytes in situ^71^ we also performed long-term studies of OCT1 regulation via different pathways^3,6,62^.

Standardized and optimized culture conditions were essential to ensure reasonable accuracy in small well format. We took several steps to this end. First, we used our new normoglycemic medium composition that ensures optimal viability, expression of enzymes and transporters, and metabolic function in the 384-well format^8^. Further, we used microplates that promote optimal spheroid formation^72^. Since we expected significant donor variability, we included 3D PHH cultures from three donors in our study^56^. Finally, we used an automated liquid handler to minimize volume variability between wells. Through these efforts, we were able to keep the variability between the single spheroids low overall. Interestingly, the interindividual variability in the kinetic parameters was smaller than expected^54,73^, especially considering the high genetic polymorphism demonstrated for OCT1^31,74,75^.

Notably, the K_m_-values of ASP+ were comparable between the three donors, indicating a high reproducibility of the experimental set-up. The accuracy of our experiments was further corroborated by the excellent agreement between kinetic experiments performed by three experimentators between 2020 and 2023 (Supplementary Table 7). These results thus convincingly demonstrate that not only drug metabolism but also the kinetics of drug transport can be studied robustly in 3D PHH with reasonable reproducibility.

Clinically important transporters such as OCT1 can be involved in drug-drug interactions (DDIs), and we therefore investigated if DDI studies could be performed in spheroids. Six drugs previously known to inhibit OCT1 were used in this study. The drugs were selected to be readily membrane permeable with log P-values ranging from 3.1 to 5.2 and a total polar surface area between 18 and 84 Å^2^ to provide good permeation into the spheroids. All six drugs inhibited the ASP+ transport to approximately the same extent. However, the inhibition was about 25% lower than previously observed in monolayers of HEK293 cells overexpressing OCT1^50^. We believe this is caused by slower penetration into the multilayered spheroids compared to the single layer of HEK cells overexpressing OCT1 used in previous screens for inhibitors. Other contributing factors to the lower inhibition could be clearance of the inhibitors via metabolism or efflux. Nevertheless, the difference between the two systems was small, and the reproducible results from our screening indicate that the PHH spheroids are applicable in predictions of transporter DDIs. Of note, incubation of 3D PHH with OCT1-inhibitors resulted in a significant decrease in ASP+ uptake detected within 5 min of assay which might facilitate the development of high-throughput screening. In addition, it was recently demonstrated that a 30 min preincubation caused significant change of IC_50_ in 21 out of 33 SLC transporter-inhibitor combinations^76^. Thus, longer incubation times might further facilitate inhibitor penetration and improve the sensitivity for high-throughput DDI testing.

The real advantage of PHH spheroid cultures relates to longer-term drug metabolism and toxicity studies, as the 3D PHH maintains a non-proliferating, differentiated phenotype for weeks in culture^3,6,8,62^. This makes it possible to study how drugs and toxic chemicals affect the transcriptome, proteome, and metabolic patterns over time and at high resolution^77^. Applications that take advantage of the long culture times include studies of cytochrome P450 enzyme induction and inhibition^62,78^, the formation of slowly produced drug metabolites that are undetectable in short-lived cultures^79^, and the investigation of toxicological readouts over days or even weeks^32^.

In the second part of this study, we therefore investigated the OCT1 expression and function response to the activation of different canonical regulatory pathways. We evaluated the effects of nine compounds previously demonstrated to have the potential to regulate nuclear receptors, induce liver injury (DILI), chronic inflammation, or steatosis after treatment for one week. Initial experiments with spheroids from donor 1 showed that only three of the compounds, the two transcription factor activators rifampicin and elafibranor and the hepatotoxic drug paracetamol (also known as acetaminophen), induced a significant approximately two-fold increase in ASP+ transport at clinically relevant concentrations. While these results were reproducible in spheroids from donor 1, these compounds did not induce OCT1 in the other two donors. Hence, for future studies additional PHH donors and extended compound libraries can be considered.

A possible explanation for the slightly increased ASP+ uptake observed in donor 1 in this study might be a mechanism beyond the change in OCT1 abundance^80^. First, synthesized transporters could be localized both in the plasma membrane or kept in the endoplasmic reticulum and Golgi, and trafficking of the transporters between these cellular compartments will affect the substrate uptake rate even if the overall protein abundance remains stable^81^. Second, common post-translational modifications (PTMs), have been demonstrated for other transporters of the SLC family^82^. Overall, hepatic transporter regulation occurs on multiple levels, from nuclear receptor signaling to mechanical modification of the protein conformation, and is of particular interest to future studies.

To ensure that the listed drugs elicited prototypic responses in all donors, we examined the proteomes resulting from each treatment. Interestingly, all three donors responded to rifampicin exposure with increased expression of CYP3A4, 3A5 and MDR1/P-glycoprotein, confirming susceptibility to activation via PXR^62,83^. Similarly, elafibranor induced enzymes involved in fatty acid oxidation in all donors, consistent with activation via PPARα/δ^67–69^. Paracetamol also altered the expression of a number of proteins, but in a random manner (Supplementary Table 5). It should be noted that a similar evaluation was not done for the additional compounds in the extended compounds set used in Fig. 3a (T0901317, chenodiol, chlorpromazine, diclofenac, TNFα, and FFA). Hence, for those compounds, we cannot fully verify that the desired 3D PHH phenotype was achieved. For instance, TNFα exposure is commonly believed to negatively affect cell state^84,85^, however there is a growing body of evidence of TNFα playing a critical role in maintaining hepatocyte homeostasis, promoting growth and long-term differentiation of 3D PHH^86^. For future more extensive studies, phenotype verification would be recommended for all compounds and we have recently suggested such a workflow for preliminary phenotypic screening of 3D PHH using global proteomics^87^.

Our results suggest that OCT1 is stably expressed and shows little difference in function between donors even after activation of signaling pathways known to affect other SLCs and metabolic enzymes in PHH. This is in contrast to the observations by others who observed a small but significant change in OCT1 mRNA levels upon exposure to inflammatory factors and rifampicin, respectively^24,88^. However, it has been repeatedly shown that mRNA levels of ADME-relevant proteins rarely correspond consistently to protein abundances^62,70^.

As previously observed, inter-donor variability had a significant impact on the variability of the global proteome (Fig. 4)^8,89^. Although our functional studies were limited to the stably expressed OCT1 transporter, it is clear that other transporters and enzymes will display donor-dependent variability. This variability is difficult to control since interindividual differences in liver physiology and status will influence the quality of different hepatocyte isolates^56,90^. Interestingly, global proteomic analysis provided new insights into the regulation of proteins in the 3D PHH liver model that could be further investigated. For example, we observed a relatively low expression of the enzyme UGT1A9 in donor 2, which could be a possible explanation for the high paracetamol sensitivity of this donor (Supplementary Fig. 4)^91–93^. Another interesting observation was that spheroids from donor 2 expressed increased levels of lipopolysaccharide-binding protein, complement factors, and plasminogen, indicating an ongoing inflammatory response possibly mediated by LPS (Supplementary Table 6). The ongoing inflammation, together with UGT1A9 deficiency, could explain the unusually high paracetamol toxicity in that donor. Moreover, other inflammation and fibrosis-associated proteins such as C-reactive protein (CRP), Serum amyloid A1 (SAA1), and collagen family IV were upregulated in donor 2 and acted as a driving component for the separation of the global proteomes in the principal component analysis (Fig. 4d)^94–96^.

In summary, our study shows that OCT1 is fully functional in 3D PHH spheroids cultured in our physiologically relevant medium. Furthermore, we show for the first time that short- and long-term studies of transporter kinetics and drug-drug interactions are feasible and highly reproducible, both in single spheroids using live microscopy and in a 384-well high-throughput format. By comparing global proteomes after different drug treatments and between different donors, insights into drug-induced activation of parallel prototypic signaling pathways and information on inter-individual differences can be obtained. Taken together, these results extend the applicability of 3D liver spheroids to the transporter domain and provide a proteomics-based approach for global studies of transporter and enzyme regulation.

## Supplementary Information

Supplementary Information 1.

Supplementary Information 2.

## Data Availability

The mass spectrometry proteomics data have been deposited to the ProteomeXchange Consortium via the PRIDE partner repository with the dataset identifier PXD045733 and is publicly available as of the date of publication. This paper does not report original code. Raw data will be made available on reasonable request from the corresponding author.
