# Supplementary material for: OCT1 (SLC22A1) transporter kinetics and regulation in primary human hepatocyte 3D spheroids
Source: Sci Rep. 2024 Jul 27;14:17334. doi: 10.1038/s41598-024-67192-6 (PMC11283471; doi:10.1038/s41598-024-67192-6)
Supplement: Supplementary file 1 — Supplementary Information 1. [file 41598_2024_67192_MOESM1_ESM.pdf]

OCT1 (SLC22A1) transporter kinetics and regulation in primary human hepatocyte 3D spheroids

Evgeniya Mickols[1a], Alina Meyer[1a], Niklas Handin[1a], Malin Stüwe[1], Jens Eriksson[2], Jakob Rudfeldt[3], Kristin Blom[3], Mårten Fryknäs[3], Mikael E. Sellin[2], Volker M. Lauschke[4,5,6], Maria Karlgren[1], Per Artursson[1*]

a These authors contributed to the manuscript equally

1 Department of Pharmacy, Uppsala University, 75123 Uppsala, Sweden

2 Science for Life Laboratory, Department of Medical Biochemistry and Microbiology, Uppsala University, Uppsala, Sweden

3 Department of Medical Sciences, Division of Cancer Pharmacology and Computational Medicine, Uppsala University, Uppsala, Sweden

4 Department of Physiology and Pharmacology, Karolinska Institute, Sweden

5 Dr Margarete Fischer-Bosch Institute of Clinical Pharmacology, Stuttgart, Germany

6 University of Tübingen, Tübingen, Germany

* corresponding author Per Artursson (Per.Artursson@farmaci.uu.se)

Supplementary Table 1. Medical and demographic information of PHH donors used in this study. Age groups: middle-age adult (31-45), old-aged adults (46-75), seniors (>75).

Donor	Sex	Age group	Diagnosis	BMI
1	F	seniors	Colorectal cancer	21
2	M	seniors	Colorectal cancer	29
3	F	Middle-age adult	Breast cancer	20

Supplementary Table 2. Chemical and physical properties of known OCT1 inhibitors.

Compound	Mol.weight g/mol	LogP	Hydrogen bind donor count	Hydrogen bind acceptor count	Rotatable bonds	Topological Polar Surface Area, $Å^2$
Ketoconazole	531.4	4.3	0	6	7	69.1
Verapamil	454.6	3.8	0	6	13	64
Clomipramine	314.9	5.2	0	2	4	6.5
Diltiazem	414.5	3.1	0	6	7	84.4
Clotrimazole	344.8	5	0	1	4	17.8
Chlorpromazine	318.9	5.2	0	3	4	31.8

Supplementary Table 3. Effect of OCT1 inhibitors.

Compound	ASP+ inhibition, % of control ± SD	
	5 min incubation	25 min incubation
Ketoconazole	52 ± 14	58 ± 12
Verapamil	48 ± 12	59 ± 11
Clomipramine	48 ± 12	52 ± 11
Diltiazem	46 ± 12	48 ± 13
Clotrimazole	35 ± 14	43 ± 13
Chlorpromazine	36 ± 13	37 ± 13

Supplementary Table 4. Chemical and physical properties of possible OCT1 modulators.

Compound	Mol.weight g/mol	LogP	Hydrogen bind donor count	Hydrogen bind acceptor count	Rotatable bonds	Topological Polar Surface Area, Å²
T0901317	481.3	4.9	1	13	5	66
Rifampicin	822.9	4.9	6	15	5	220
Chenodiol	392.6	4.9	3	4	4	77.8
Elafibranor	384.5	5.1	1	5	7	88.9
Chlorpromazine	318.9	5.2	0	3	4	31.8
Paracetamol	151.16	0.5	2	2	1	49.3
Diclofenac	296.1	4.4	2	3	4	49.3

Supplementary Table 5. List of the proteins that are upregulated in the profile plots in Figure 4 and Supplementary Figure 1. The profiles of the upregulated proteins were retrieved based on the compound exposure variable.

Compound	Gene name	Protein
DMSO<br><br>*No enrichment in any of the pathways or protein clustering detected*	DIP2B	Disco-interacting protein 2 homolog B
	DYNC1LI2	Cytoplasmic dynein 1 light intermediate chain 2
	ENAH	Protein enabled homolog
	FAM184B	Protein FAM184B
	HSD3B7	3 beta-hydroxysteroid dehydrogenase type 7
	ITGA2	Integrin alpha-2
	PABPC1	Polyadenylate-binding protein 1
	PDIA5	Protein disulfide-isomerase A5
	RAB8A	Ras-related protein Rab-8A
	RTCA	RNA 3-terminal phosphate cyclase
Elafibranor<br><br>*Significant enrichment of fatty acid beta activation pathway, FDR value 4.09E-4*	AADAC	Arylacetamide deacetylase
	ACADM	Medium-chain specific acyl-CoA dehydrogenase, mitochondrial
	ACADVL	Very long-chain specific acyl-CoA dehydrogenase, mitochondrial
	CLUH	Clustered mitochondria protein homolog
	CPT1A	Carnitine O-palmitoyltransferase 1, liver isoform
	ECH1	Delta(3,5)-Delta(2,4)-dienoyl-CoA isomerase, mitochondrial
	FABP1	Fatty acid-binding protein, liver
	HSD17B4	Peroxisomal multifunctional enzyme type 2
Paracetamol<br><br>*No enrichment in any of the pathways or protein clustering detected*	ACTL6A	Actin-like protein 6A
	CYP2A7	Cytochrome P450 2A7
	EXOSC8	Exosome complex component RRP43
	GSTM4	Glutathione S-transferase Mu 4
	HCFC1	Host cell factor 1
	INSR	Insulin receptor
	LMAN2L	VIP36-like protein
	NOP58	Nucleolar protein 58
	OAT	Ornithine aminotransferase
	TATDN1	Putative deoxyribonuclease TATDN1
Rifampicin<br><br>*No enrichment in any of the pathways, clustering of drug metabolising enzymes*	CYP3A4	Cytochrome P450 3A4
	CYP3A5	Cytochrome P450 3A5
	MDR1	Multidrug resistance protein 1 or ATP-binding cassette subfamily B member 1

Supplementary Table 6. List of the proteins that are upregulated in the profile plots in Figure 4 and Supplementary Figure 1. The profiles of the upregulated proteins were retrieved based on the donor variable

Donor	Gene	Protein
Donor 1 *No enrichment in any of the pathways or protein clustering detected*	ADH1B	Alcohol dehydrogenase 1B
	ALDOC	Fructose-bisphosphate aldolase C
	DDC	Aromatic-L-amino-acid decarboxylase
	GM2A	Ganglioside GM2 activator;Ganglioside GM2 activator isoform short
	GSTT1	Glutathione S-transferase theta-1
	HMGCR	3-hydroxy-3-methylglutaryl-coenzyme A reductase
	LRRFIP1	Leucine-rich repeat flightless-interacting protein 1
	LTA4H	Leukotriene A-4 hydrolase
	OAS2	2-5-oligoadenylate synthase 2
	UBA1	Ubiquitin-like modifier-activating enzyme 1
Donor 2 *Detection of molecule of bacterial origin clustering, no significant enrichment*	ALDH16A1	Aldehyde dehydrogenase family 16 member A1
	C1S	Complement C1s subcomponent
	C4B	Complement C4-B
	CALML5	Calmodulin-like protein 5
	FGL1	Fibrinogen-like protein 1
	H2AFY2	Core histone macro-H2A.2
	LBP	Lipopolysaccharide-binding protein
	NNMT	Nicotinamide N-methyltransferase
	PLG	Plasminogen
	TLN1	Talin-1
Donor 3 *Cellular detoxification of nitrogen compounds clustering, no enrichment*	ANXA6	Annexin A6
	APOA5	Apolipoprotein A-V
	C14orf159	UPF0317 protein C14orf159, mitochondrial
	ENO3	Beta-enolase
	GLS2	Glutaminase liver isoform, mitochondrial
	GLYATL1	Glycine N-acyltransferase-like protein 1
	GSTM1	Glutathione S-transferase Mu 1
	GSTM3	Glutathione S-transferase Mu 3
	LETM1	LETM1 and EF-hand domain-containing protein 1, mitochondrial
	SULT1A1	Sulfotransferase 1A1

Supplementary Table 7. Michaelis-Menten kinetics for donor 1 performed in 2020, 2021 and 2023.

Year	Apparent Km, µM	Apparent Vmax, RFU/min
2020	17.4	346.7
2022	15.7	648.9
2023	16.6	228.8

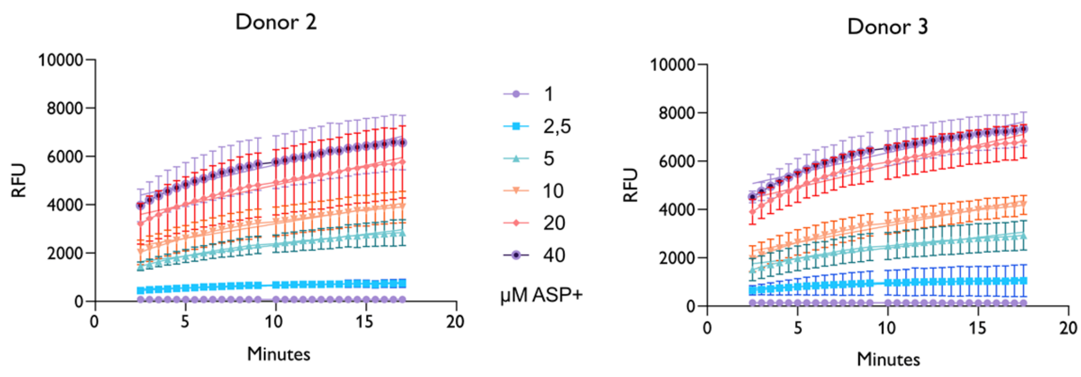

Supplementary Figure 1. Linear uptake interval (0 to 17 minutes) was used to calculate Km and Vmax in 3D PHH from donors 2 and 3 (n=8-10).

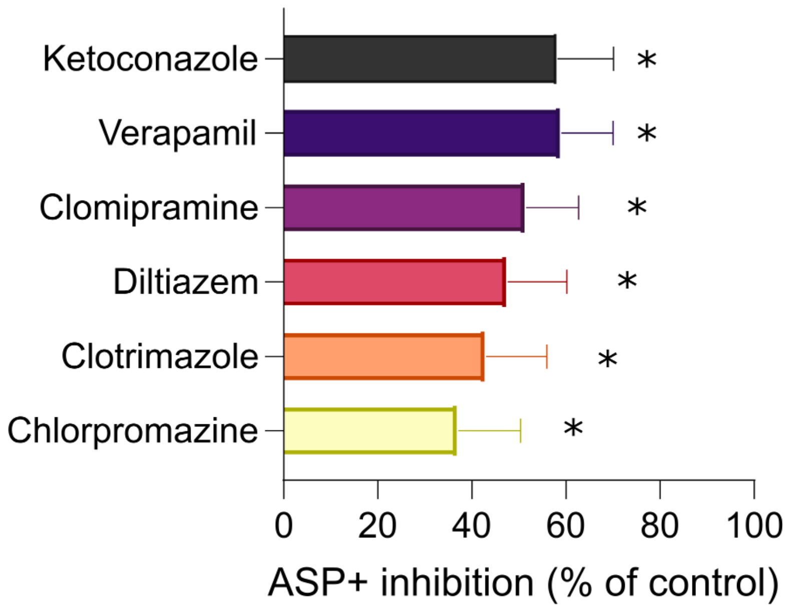

Supplementary Figure 2. Uptake of ASP+ (1 µM) was measured after 25 minutes in the presence of 100 µM of the OCT1 inhibitors. Inhibitory effect of compounds is shown as percentage of vehicle control, n=8-12. All observations were statistically significant in multiple unpaired t-tests compared to vehicle control.

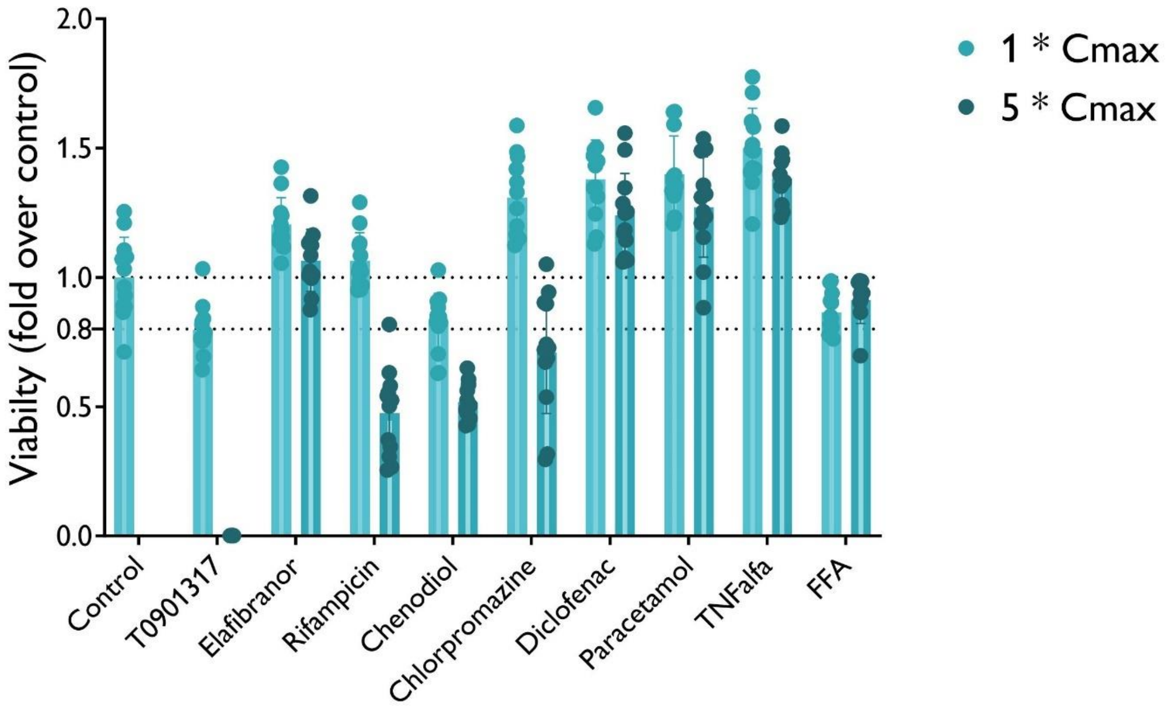

Supplementary Figure 3. Viability data for all compounds at 1 * Cmax and 5 * Cmax in donor 1. Compounds failing viability threshold (<0.8): T0901317, Rifampicin, Chenodiol, Chlorpromazine.

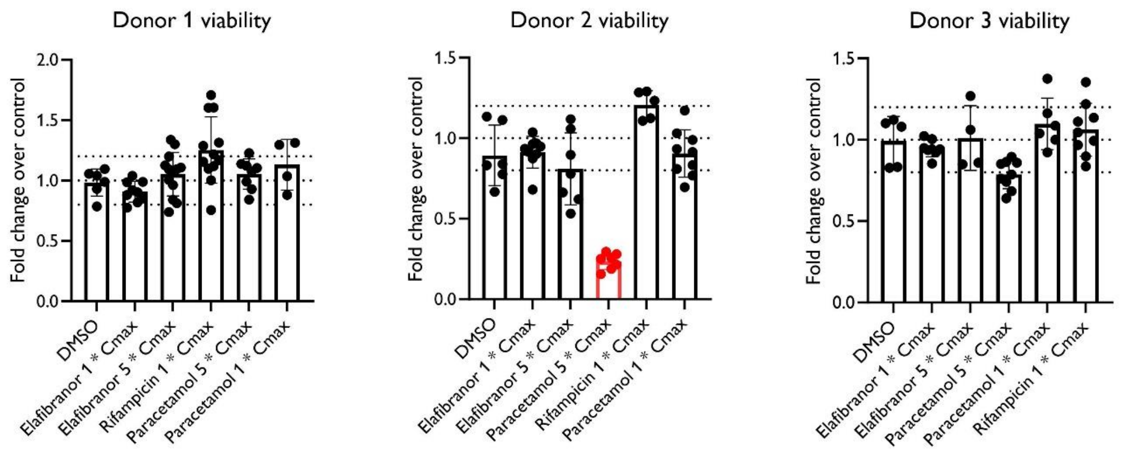

Supplementary Figure 4. Viability data for all compounds in three donors at 1 * Cmax and 5 * Cmax in donor 1. Condition failing viability threshold (<0.8) is highlighted in red.

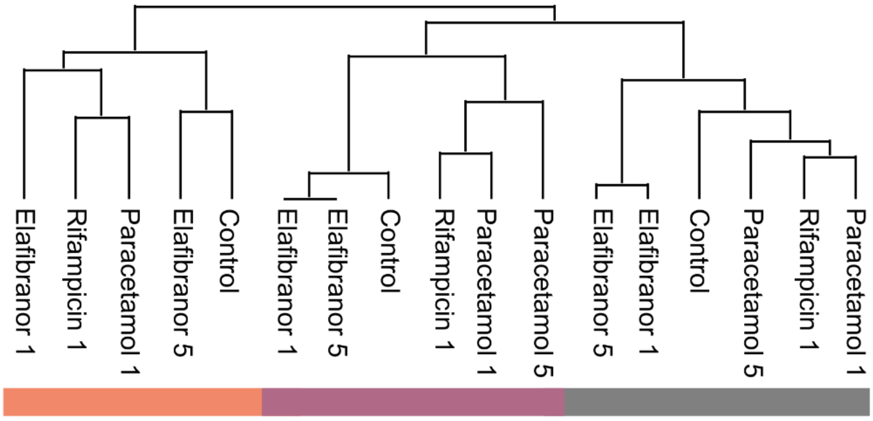

Supplementary Figure 5. In the hierarchical clustering of 3D PHH proteomes, branch length is scaled to represent Euclidean distance. Numbers 1 or 5 represent exposure to one and five times the Cmax concentration, respectively.

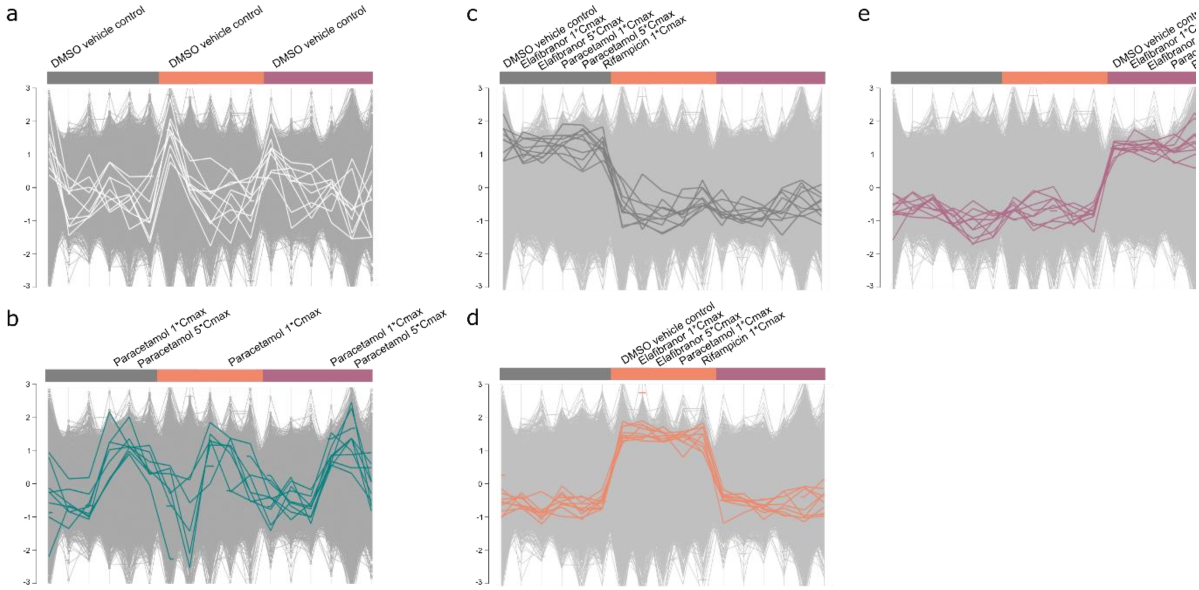

Supplementary Figure 6. Profile plots of proteins upregulated based on the compound exposure variable a) vehicle control DMSO 0.025%, b) paracetamol; or donor factor c) donor 1, d) donor 2, e) donor 3

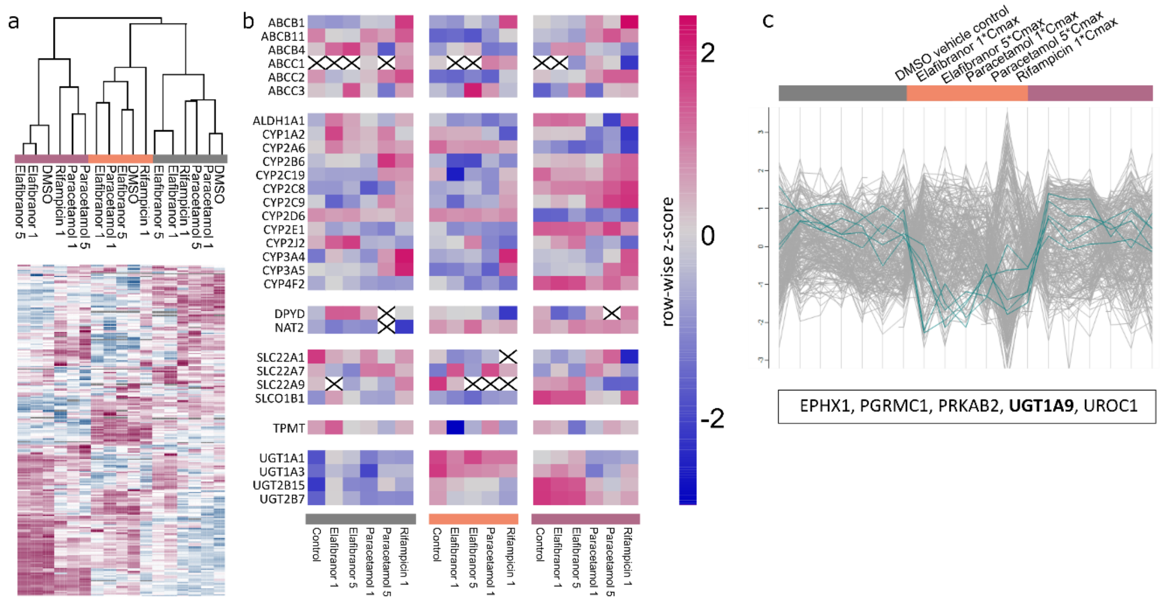

Supplementary Figure 7. Expression of the ADMET proteins in the studied samples. a) Hierarchical clustering of the ADME proteins in 3D PHH, branch length is scaled to represent the Euclidean distance. The numbers 1 or 5 indicate exposure to one or five times Cmax concentration, respectively. b) Z-score normalized heatmap of clinically important ADMET protein expression. c) Profile plots of ADMET proteins downregulated in the donor 2.
